# Multi-Resonant Indolo[3,2,1-*jk*]carbazole-Based Host for Blue Phosphorescent Organic Light-Emitting Diodes

**DOI:** 10.3390/molecules28135118

**Published:** 2023-06-29

**Authors:** Xiang Li, Peng Yuan, Jinyu Song, Yu Chang, Xueting Jiao, Jianfen Zhao, Cong Zhang, Wenjuan Li, Xiao-Chun Hang

**Affiliations:** 1 School of Flexible Electronics (Future Technologies), Nanjing Tech University, 30 South Puzhu Road, Nanjing 211816, China; 2 Nanjing Jianuolin Optoelectronics Technology Co., Ltd., 5th Xin Mofan Road, Nanjing 210009, China

**Keywords:** indolo[3,2,1-*jk*]carbazole, multi-resonance effect, blue PhOLED, host

## Abstract

In organic light-emitting diodes, positive and negative charge carriers mostly migrate at different rates. This could result in excitons formed in the EML often migrating to the vicinity of the hole transport layer and the electron transport layer. To address this, it is important to design high-quality multi-resonance hosts that can balance the migration rate of carriers. Here, we report two newly developed multi-resonance hosts, m-ICzPBI and o-ICzPBI. The hosts contain an indolo[3,2,1-*jk*]carbazole (ICz) motif, which functionalized as either a donor or an acceptor unit. The hosts exhibit extremely high molecular rigidity and thermal stability. Devices A and B were constructed using FIrpic as a phosphorescent emitter with m-ICzPBI or o-ICzPBI as a host. Device A achieved high maximum values of EQE, PE and CE of 13.4%, 24.8 lm W^−1^ and 31.6 cd A^−1^, respectively, and low efficiency roll-off at 5000 cd m^−2^ of 8.6%, 10.6 lm W^−1^ and 20.3 cd A^−1^, respectively.

## 1. Introduction

Organic light-emitting diodes (OLEDs) have been developed rapidly in recent years due to their important role in the late-model application of lighting and display [1]. Phosphorescent OLEDs (PhOLEDs) are a type of OLED that can utilize both singlet and triplet excitons. By using both types of excitons, PhOLEDs can achieve an internal quantum efficiency as high as 100%, making them the most efficient OLEDs [2,3]. Mostly, a phosphorescent emitter is uniformly doped in the host material at a low concentration. At high concentrations, quenching can occur, which significantly reduces the luminescent efficiency and stability for PhOLEDs [4]. Therefore, studying host materials is one of the key factors for improving the performance of PhOLEDs.

**Scheme 1:**
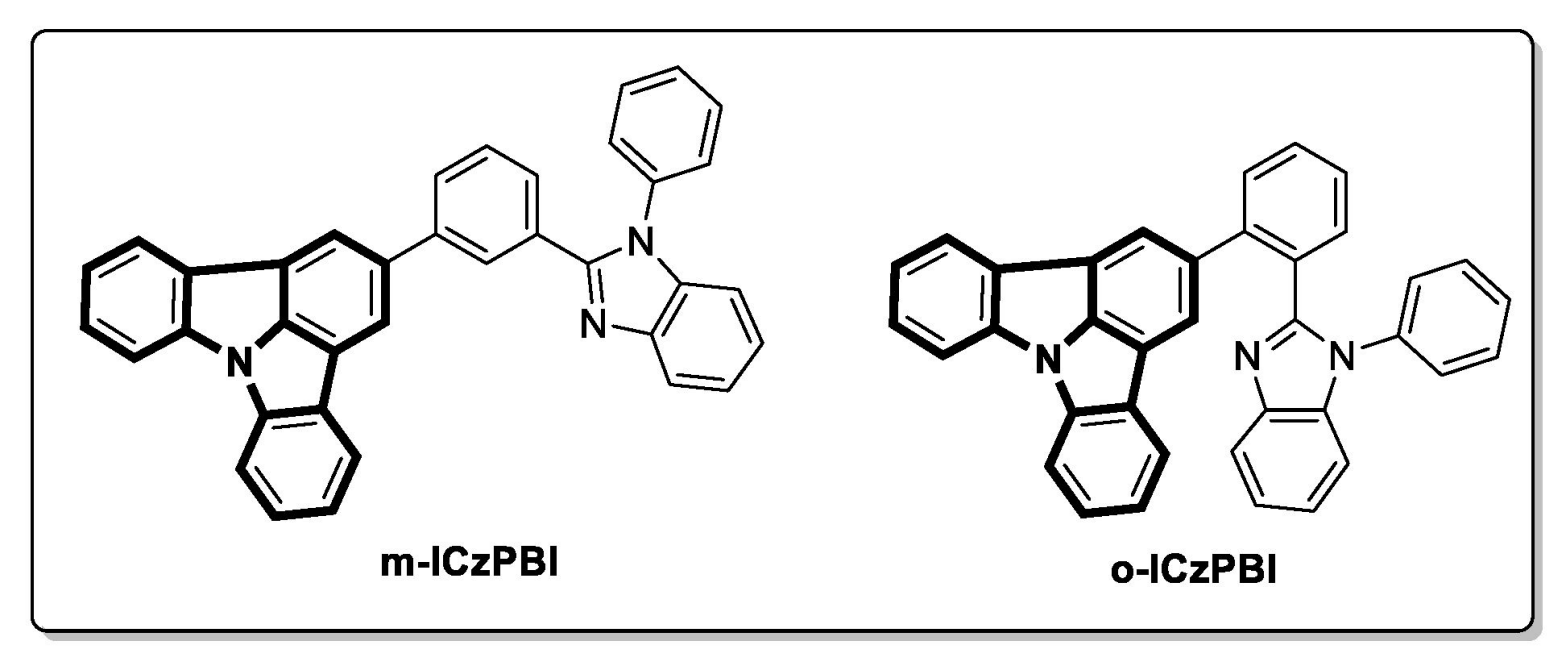
Multi-resonance host molecules.

Basically, constructing efficient PhOLEDs requires matching the energy levels as well as the lowest triplet energy (*E*_T1_) of the host–guest system in the emissive layers [5]. A host with multi-resonance properties, which can transport holes and electrons in a relatively balanced manner, is developed for PhOLEDs. In those PhOLEDs, the recombination region is broadened, which ultimately improves performance with high efficiency [6], extends the working lifetime [7] and gives optimum color purity [8]. Indolo[3,2,1-*jk*]carbazole (ICz) is widely investigated as an excellent acceptor of electro-luminescent function materials, such as thermally activated delayed fluorescence (TADF) emitters and sensitizers [9,10,11,12,13,14,15,16,17,18,19,20,21]. However, it is rarely considered as a host despite its excellent hole and electron transport capabilities due to its multi-resonance property [18]. Moreover, the ICz motif has high *E*_T_ energy and thermal stability, making it a suitable blue host for PhOLEDs. Here, we synthesized and reported two new multi-resonance host materials, 2-(3-(1-phenyl-1*H*-benzo[*d*]imidazol-2-yl)phenyl)indolo[3,2,1-*jk*]carbazole (m-ICzPBI) and 2-(1-phenyl-1*H*-benzo[*d*]imidazol-2-yl)phenyl)indolo[3,2,1-*jk*]carbazole (o-ICzPBI), with ICz as either the donor or the acceptor (Scheme 1). The material 1-Phenyl-1*H*-benzimidazole (PBI) is conventionally used as an acceptor [22], which can enhance the electron injection/transport ability of materials, and its larger structural unit is also beneficial for improving the thermodynamic properties of materials [23]. Their thermal, electronic and photoluminescent properties were fully investigated through both computational analysis and experimental characterization. Single-carrier devices based on m-ICzPBI and o-ICzPBI were constructed to verify the balance of carrier transport. Finally, blue PhOLEDs using m-ICzPBI or o-ICzPBI as a host doped with iridium bis(4,6-difluorophenypyridinato-N,C2′) picolinate (FIrpic) were fabricated, and achieved maximum external quantum efficiency (EQE_max_), maximum power efficiency (PE_max_) and maximum current efficiency (CE_max_) of 13.4%, 24.8 lm W^−1^ and 31.6 cd A^−1^, respectively, and also showed low roll-off.

## 2. Results and Discussion

### 2.1. Theoretical Calculation

**Figure 1:**
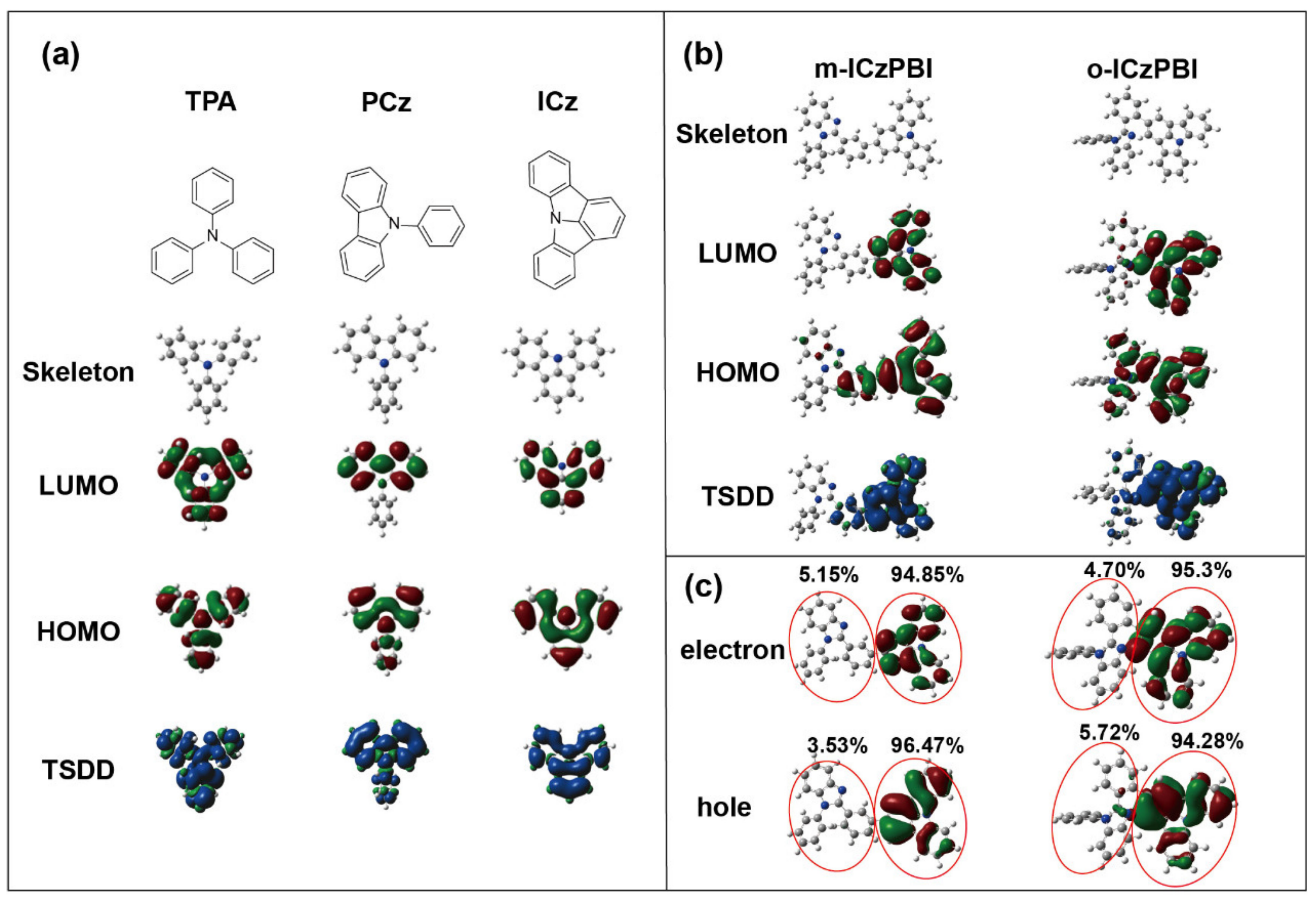
Computational simulation. HOMO/LUMO/TSDD distributions (**a**,**b**) and hole–electron distributions (**c**).

B3LYP/6-31G(d) is employed to investigate molecular structure and photoelectric properties. As shown in Figure 1a, the basic triphenylamine (TPA) structure has a planar geometry, but phenyls groups twist and intersect with each other at certain angles (41.65°). The lowest unoccupied molecular orbital (LUMO) of TPA is distributed on three benzene rings, while the highest occupied molecular orbital (HOMO) is distributed throughout the entire molecule. The N-phenyl carbazole (PCz) molecule is also in a quasi-planar geometry. The carbazole portion is in a planar geometry while there is a twisting angle between the benzene ring and the carbazole plane of 56.02°. The LUMO of PCz is distributed on the carbazole, while the HOMO is distributed throughout the entire molecule. The ICz unit has a completely planar structure, with evenly distributed LUMO/HOMO across the entire molecule, illustrating a multi-resonance effect [19,24]. Compared with TPA and PCz, the spin density distribution of the ICz triplet is more uniform across the entire molecule, likely due to the presence of bonded phenyls with better conjugation. However, increased electronic delocalization will lead to lowering T_1_ energy. The triplet state energy levels of TPA, PCz and ICz are 3.18 eV, 3.18 eV and 2.98 eV, respectively, according to the simulation calculation of density functional theory (DFT) (Table 1). To be noted, as the molecular rigidity increases, their HOMO energy levels decrease sequentially, implying ease of hole injection and transport. Meanwhile, their LUMO energy levels also decrease correspondingly, which indicates significant enhancement in electron injection and transport for ICz with a larger decrease in value. Generally, electron mobility is orders of magnitude lower than hole mobility. With increased molecular rigidity and electron mobility, ICz is capable of achieving a hole–electron balance in the exciton recombination and carrier transporting processes. In addition, the good planarity of the ICz motif also can enhance the thermal stability of molecules as well as the working stability of devices, although it may reduce the energy gap. The lowest triplet energy (T_1_) of ICz is calculated to be 2.98 eV, which is close to the energy of standard blue light (2.70 eV). Noteworthy, compared to TPA and PCz, the T_1_ energy of ICz is decreased by about 0.1~2 eV less than that of the lowest singlet energy (S_1_), which can be attributed to the multi-resonate effect that minimizes the gap between *E*_S1_ and *E*_T1_. Therefore, the molecule with an ICz motif should be carefully managed by minimizing the reduction of *E*_T_ to meet the basic requirements for blue PhOLEDs. Molecular twisting and conjugating restriction can be considered when using ICz with other ancillary units for constructing blue hosts. It may be possible to combine all desired merits while maintaining the high *E*_T1_ needed for blue PhOLEDs.

As shown in Figure 1b, both m-ICzPBI and o-ICzPBI molecules have twisted molecular structures. There are twist angles between the flat carbazole fragment of ICz and both phenyl spacer units (40.56° and 54.94°). The twisted structure limits its conjugation length, thereby retaining high E_T_ energy within the molecule. Such a distorted molecular structure can also hamper electron transfer between bonded molecular moieties and intermolecular interreactions [1]. These properties improve stability in a solid matrix by reducing the tendency of crystallization. The LUMO orbitals of m-ICzPBI are distributed in the ICz part, while the HOMO orbitals are distributed in the ICz and spacer phenyl units. The result indicates that the PMI motif has weak electron injection ability. Similarly, the LUMO orbitals of o-ICzPBI are mainly distributed in the ICz part, and the HOMO orbitals are distributed in the PMI part. Upon comparison, it is clear that o-ICzPBI with a meta-position connection exhibits better conjugation. Spin density distributions (TSDDs) were performed to investigate the T_1_ of TPA, PCz, ICz, m-ICzPBI and o-ICzPBI. The TSDD of ICz is uniformly distributed across the whole molecular surface, indicating a multi-resonate effect for triplet transition. The TSDDs of m-ICzPBI and o-ICzPBI are concentrated on the ICz moiety, with a small extension to the phenyl spacer and PMI. Natural transition orbitals (NTO) analysis, based on the geometry of T_1_, illustrates that the T_1_ → S_0_ transition mostly occurred within the ICz moiety. The results indicate that both m-ICzPBI and o-ICzPBI maintained high *E*_T1_ of ICz, and therefore are suitable to function as hosts for blue PhOLEDs.

### 2.2. Synthesis and Structure Characterization

**Scheme 2:**
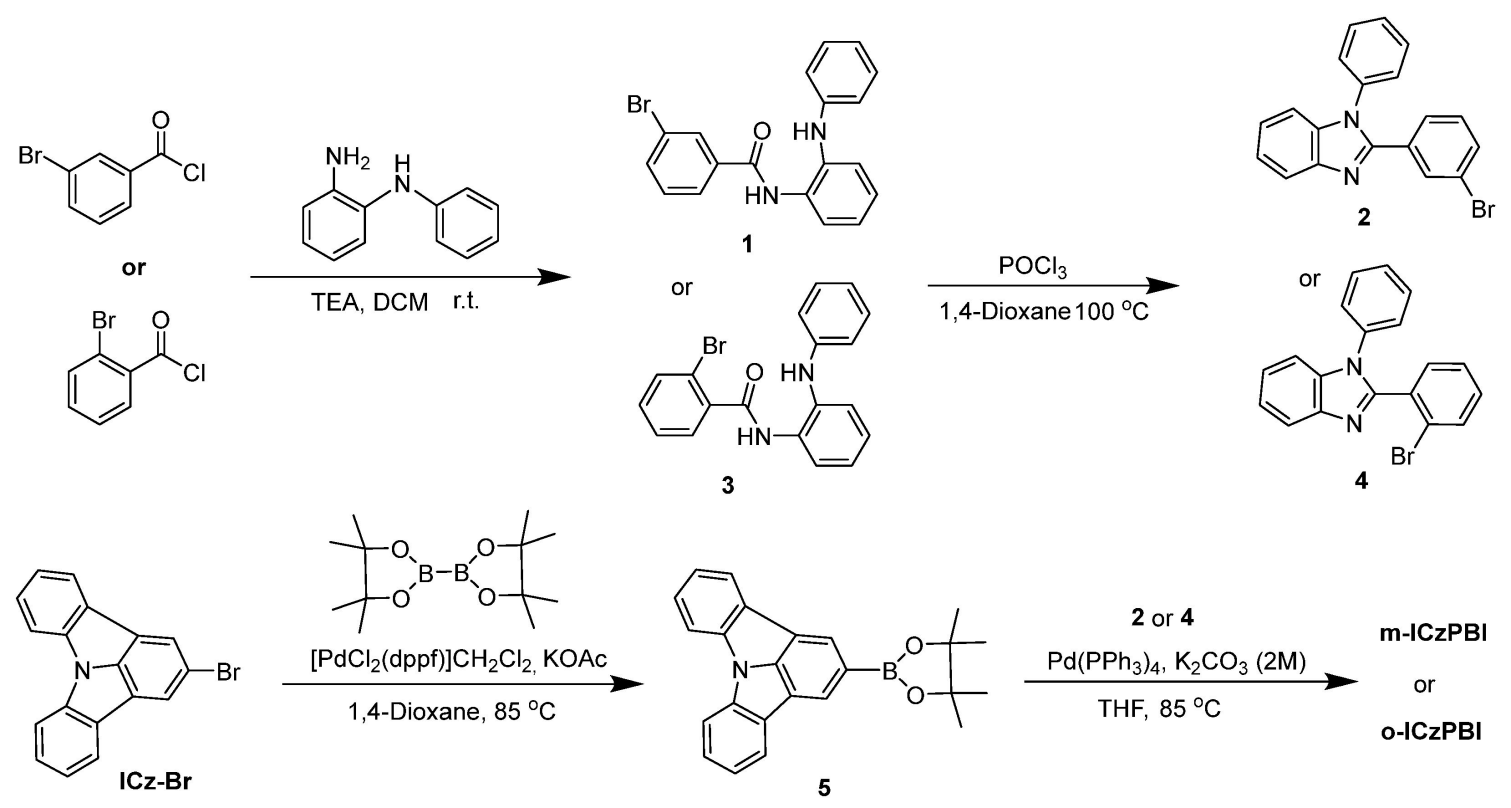
Synthetic routes of m-ICzPBI and o-ICzPBI.

m-ICzPBI and o-ICzPBI were prepared by palladium-catalyzed cross-coupling reactions of the phenyl PMI bromide segment (**2** and **4**) and ICz boronic unit (**5**) in good yields (Scheme 2). Compounds **2** and **4** were synthesized by an imidazolyl ring-closure reaction, whereas boronic compound **5** was transferred from its bromide substituent (ICz-Br). All the starting materials are commercially available. The newly synthesized compounds were conventionally characterized via ^1^H NMR, ^13^C NMR spectra and Liquid Chromatograph Mass Spectrometer (LC-MS). The chromatographically purified products were further sublimated at 250 ℃ under a pressure of 1 × 10^−4^ Pa to obtain high-grade purified materials suitable for device fabrications. Please refer to Synthesis and Structure Characterization for the specific synthesis process.

### 2.3. Thermal Properties

Thermal stability of molecules of TPA, PCz, ICz, m-ICzPBI and o-ICzPBI was performed by thermogravimetric analysis (TGA) under an N_2_ atmosphere, which showed the thermal decomposition temperature (*T*_d_) of 5% weight loss at 219, 251, 337, 464 and 408 °C respectively (Figure S7). Compared to TPA and PCz, ICz exhibits enhanced rigidity leading to higher *T*_d_. Both m-ICzPBI and o-ICzPBI have extremely high *T*_d_ due to their highly rigid π moieties of ICz, PMI and phenyl spacer. These results indicate that m-ICzPBI and o-ICzPBI are highly thermally stable for OLED fabrication [25,26]. Glass transition temperatures (*T_g_*) and melting temperatures (*T*_m_) of m-ICzPBI and o-ICzPBI were measured by a differential scanning calorimeter (DSC), and were found to be almost identical, at 234 ± 1 and 114 ± 1 °C, respectively. It can be seen that the substitution method of meta or ortho sites has little effect on *T_g_* and *T*_m_ of these two materials (Figure S8). These values are significantly higher than commonly used hosts, such as CBP (4,4′-Bis(N-carbazolyl)-1,1′-biphenyl, 62 °C), SimCP (9,9′-(5-(triphenylsilyl)-1,3-phenylene)bis(9H-carbazole), 101 °C), etc., indicating superb phase stability [27].

### 2.4. Photophysical Properties

**Figure 2:**
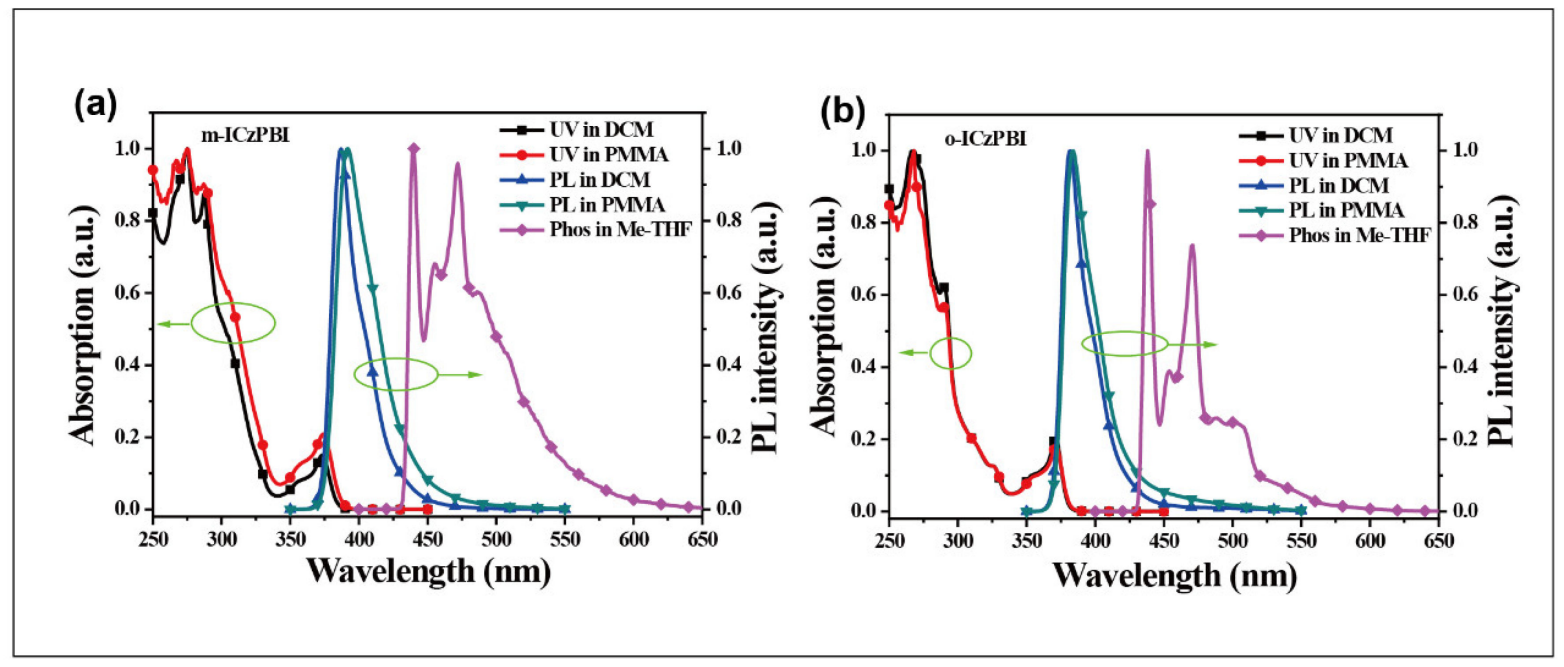
UV–vis absorption and photoluminescence of m-ICzPBI (**a**) and o-ICzPBI) (**b**). Samples in DCM and 5%-doped PMMA were measured at RT, and phosphorescent emissions were measured at 77 K in 2Me-THF solution.

Absorption and emission spectra are comparatively shown in Figure 2, while photophysical data corresponding to the peak wavelength, energy values of the frontier molecular orbitals (FMOs), etc., are summarized in Table 2. Due to the predominant distribution on ICz of FMOs as well as electronic transition, m-ICzPBI and o-ICzPBI show relatively similar absorption and emission spectra as well as parameters. Absorption peaks below 300 nm are attributed to the π-π* transition of phenyl spacer and PMI, while the broad absorption bands at approximately 350~390 nm are the characteristic absorption of ICz [14]. The energies of the lowest singlet (*E*_S1_) of m-ICzPBI and o-ICzPBI, calibrated by the intersection of absorption and emission in dichloromethane (DCM) at an ambient condition, are 3.21 eV and 3.25 eV, respectively. m-ICzPBI and o-ICzPBI show a narrow-spectra emission in the near-ultraviolet to blue region with peaks at 382~392 nm [20]. The full widths of half maximum of the emission spectra of m-ICzPBI and o-ICzPBI in DCM are measured at 25 and 23 nm, respectively. It can be seen that the redshift in the emission peak of m-ICzPBI is more obvious. The triplet state energy (*E*_T_) levels of m-ICzPBI and o-ICzPBI are both 2.83 eV, calculated by the phosphorescence spectra at 77 K temperature, and meet the basic requirements for blue light energy (*E*_T_ > 2.70 eV). The results indicate that m-ICzPBI and o-ICzPBI can be used as host materials of blue PhOLEDs. The singlet-state energy level S1 and triplet-state energy level T1 of the material can be calculated based on the emission spectrum at room temperature and the position of the first emission peak in the low-temperature 77 K phosphorescence spectrum. Their HOMO and LUMO energy levels were calculated from the onset of oxidation (*E*_ox_) and reduction potentials (*E*_re_) according to the equation E_HOMO/LUMO_ = −[*E*_ox/re_ − *E*_(Fc/Fc_^+^_)_ + 4.8] eV, respectively. Redox or oxidation potentials are reported referring to ferrocenium/ferrocene (Fc^+^/Fc) (Figure S9). The HOMO–LUMO values achieved from CV measurements are significant; however, more accurate values in solid state devices can refer to ionization and electron affinity potentials, which show lower values for m-ICzPBI and o-ICzPBI in a solid state.

### 2.5. Electroluminescent Properties

**Figure 3:**
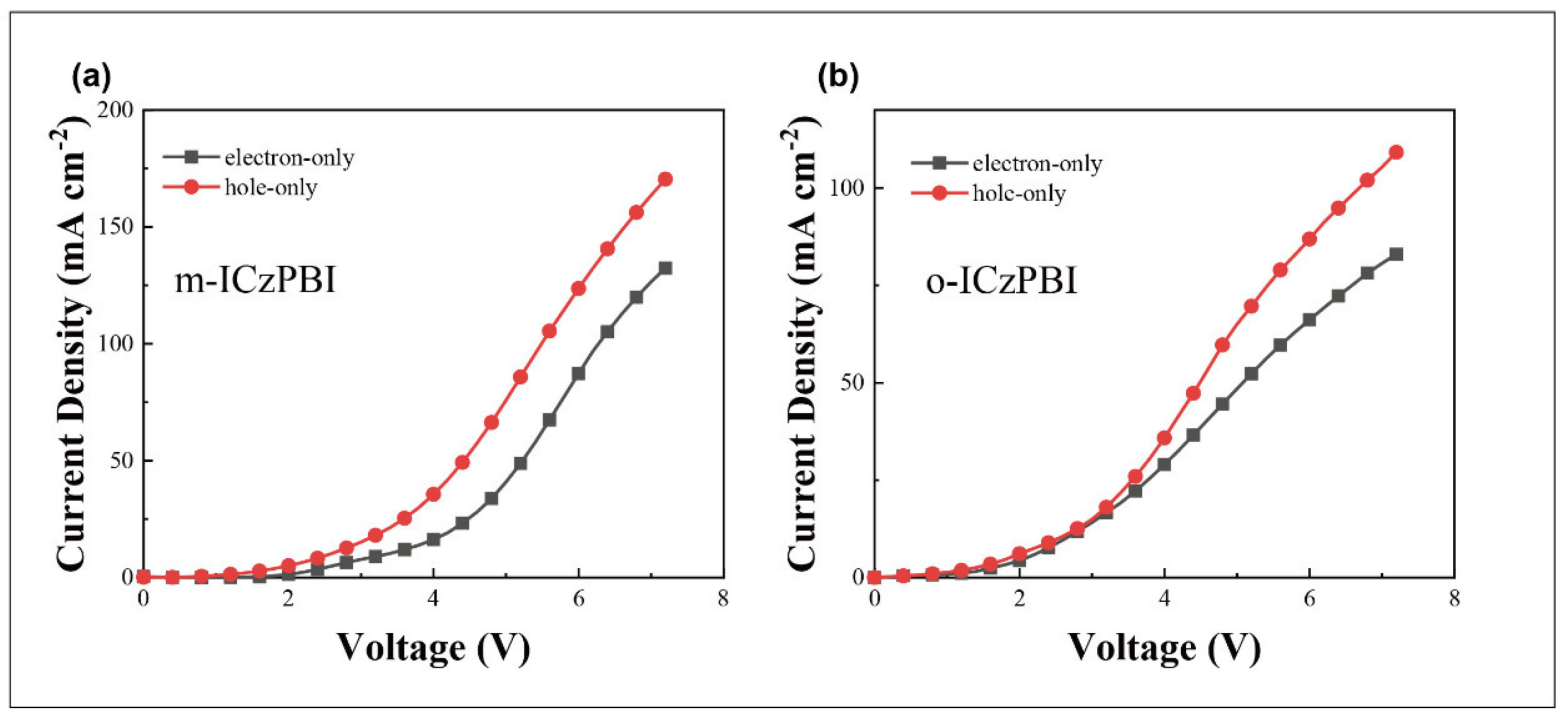
The current density–voltage (J–V) curves for electron- and hole-only devices for m-ICzPBI (**a**) and o-ICzPBI (**b**).

Charge transfer characteristics play a crucial role in the performance of OLED devices. To examine the charge transport characteristics of m-ICzPBI and o-ICzPBI, electron- and hole-only devices were fabricated with the following structures: ITO/Liq (10 nm)/host (50 nm)/Liq (10 nm)/Al (120 nm) and ITO/HATCN (10 nm)/host (50 nm)/HATCN (10 nm)/Al (120 nm), respectively (Figure 3). Preliminary evaluation of carrier mobility in semiconductors using the space charge limited current (SCLC) theoretical formula gives the formula J = 9ε_r_ε_0_µE^2^/8L. ε_r_ is the relative dielectric constant, and its value is three for organic compounds. ε_0_ is the permittivity of a vacuum (8.85 × 10^−12^ F m^−1^), and L and E are the thickness of the organic layer and the electric field, respectively [28]. We performed square-root processing on the vertical axis and fit the curve, and the slope of the obtained straight line was the relevant value of carrier mobility [29]. An approximate value for single carrier mobility can be obtained by squaring the obtained slope value of the straight line and dividing it by the relevant constants.

Compared to o-ICzPBI, m-ICzPBI shows higher current density under the same driving voltage, implying better charge transport capability as well as balance that could lead to a wider exciton recombination region (Figure S11). According to the formula, the HOMO energy level of m-ICzPBI is −5.81 eV and the LUMO energy level is −2.17 eV, while the HOMO energy level of o-ICzPBI is −5.68 eV and the LUMO energy level is −2.18 eV. The lower HOMO–LUMO energy levels for m-ICzPBI indicate that the material has both good electron donating and hole transporting ability. This is in accordance with the simulated results for ICz, showing the advantage of balanced carrier mobility.

**Figure 4:**
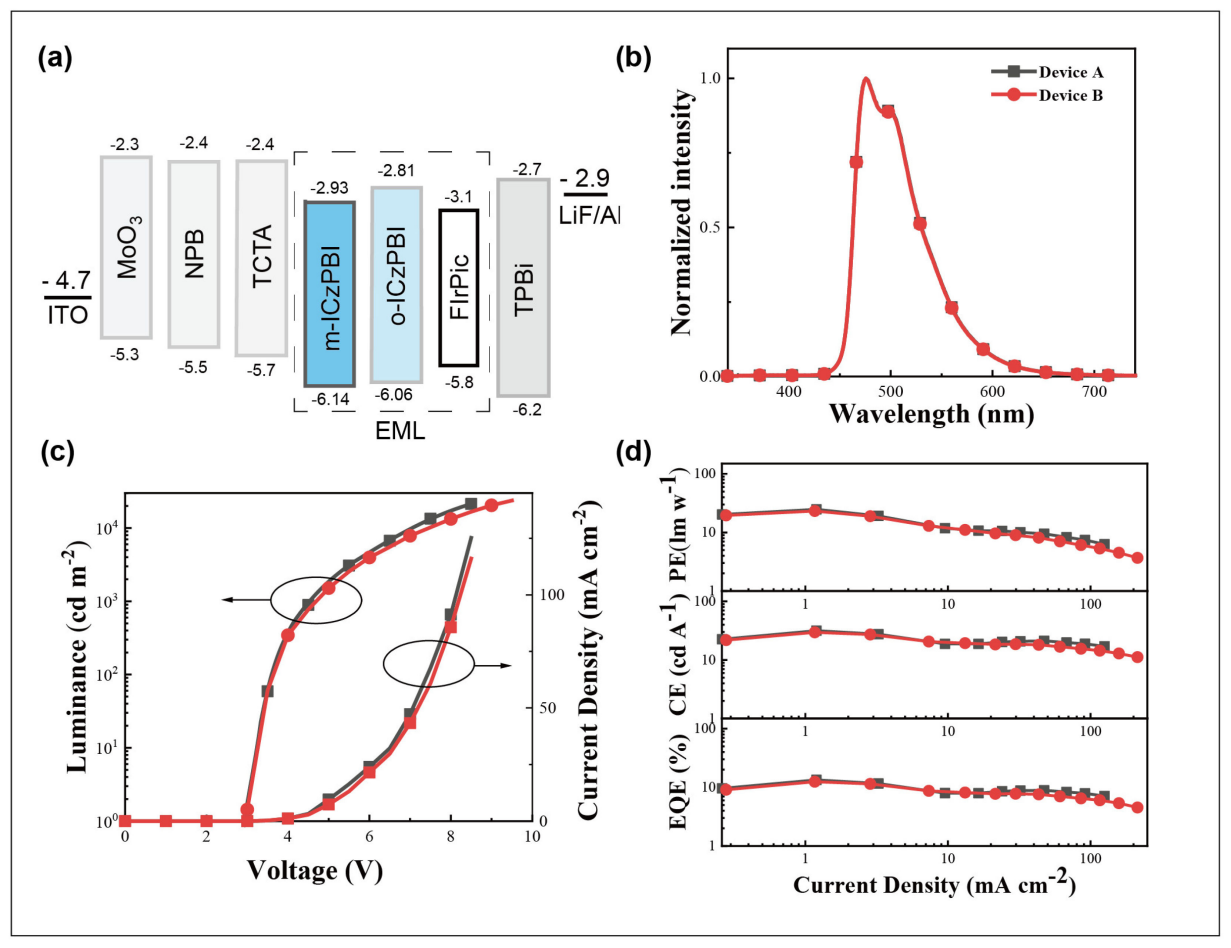
PhOLEDs. (**a**) Device architectures; (**b**) electroluminescent spectra; (**c**) I−V−L curves; (**d**) EQE−I, PE−I and CE−I curves.

The host materials we designed, m-ICzPBI and o-ICzPBI, have good thermal stability and film-forming properties. Their HOMO/LUMO energy levels are relatively suitable, with a wide band gap, which can cover the energy levels of the guest material, and their *E*_T_ is as high as 2.83 eV, making them suitable as the host materials for blue PhOLEDs. PhOLEDs utilizing FIrpic as a blue emitter were prepared to investigate the luminescent performance of m-ICzPBI and o-ICzPBI as hosts [30]. The device structure, energy level diagram and chemical structures of the functional materials of blue PhOLEDs are schematically shown in Figure 4 and Figure S10 [31,32,33]. The device architecture is ITO/MoO_3_ (3 nm)/NPB (30 nm)/TCTA (10 nm)/EML/TPBi (35 nm)/LiF (1 nm)/Al (10 nm). In the device structure, MoO_3_, NPB, TPBi and LiF function as hole injection, hole transport, electron transport and electron injection layers, respectively, to effectively reduce the hole and electron injection barrier, resulting in a lower turn-on voltage for the device. NPB has a lower triplet energy of 2.3 eV than that of the guest, which will quench excitons [34]. Thus, a TCTA with a higher triplet energy of 2.85 eV is added to block charge quenching between host and NPB, which is expected to achieve high performance.

The performance of devices A and B, with respect to the host m-ICzPBI and o-ICzPBI, is shown in Figure 4, and the related data are collected in Table 3. Both devices A and B exhibit typical blue emission spectra peaking at 476 nm, indicating full energy transfer from the host to FIrpic. The devices bear a low turn-on voltage of 3 eV, and achieve high luminance over 21,000 cd m^−2^. Devices of ICzPBI and o-ICzPBI demonstrate good electroluminescent performance with maximum external quantum efficiency (EQE_max_), power efficiency (PE_max_) and current efficiency (CE_max_) of 13.4%, 24.8 lm W^−1^ and 31.6 cd A^−1^, respectively, for device A and 12.5%, 24.2 lm W^−1^ and 29.5 cd A^−1^ for device B, respectively. Device A of m-ICzPBI shows higher efficiency values than those of device B of o-ICzPBI, which is consistent to its better charge transport capability. Additionally, the Commission Internationale de L’Eclairage (CIE) values for both are 0.17 and 0.40. Moreover, at 1000 cd m^−1^, device A still has EQE, CE and PE at 11.2 cd A^−1^, 18.3 cd A^−1^ and 26.3 lm W^−1^, respectively, exhibiting low efficiency roll-off. Recalling the electronic transition properties of m-ICzPBI and o-ICzPBI, m-ICzPBI with a less conjugated structure is likely to be better as a host due to a larger energy gap and higher charge motilities.

## 3. Materials and Methods

### 3.1. Structure Characterization

^1^H NMR spectra were recorded at 400 MHz on NMR instruments in chloroform-*d* or DMSO-*d_6_* solution; ^13^C NMR spectra were recorded at 126 MHz on Varian Liquid-State NMR instruments in chloroform-*d* solution. Mass spectra were recorded on a liquid chromatograph mass spectrometer (LC-MS).

### 3.2. Electrochemical Analysis

Cyclic voltammetry was performed using a CH Instrument 660E electrochemical analyzer under a nitrogen atmosphere in a glovebox. Anhydrous DMF was used as the solvent for reduction and oxidation, respectively. Some 0.1 M tetra(*n*-butyl) ammonium hexafluorophosphate was used as the supporting electrolyte. A silver wire was used as the pseudo reference electrode, a Pt wire was used as the counter electrode, and a platinum column was used as the working electrode. Redox or oxidation potentials are reported referring to ferrocenium/ferrocene (Fc^+^/Fc). Their HOMO and LUMO energy levels were calculated from the onset of oxidation (*E*_ox_) and reduction potentials (*E*_re_) according to the equation E_HOMO/LUMO_ = −[*E*_ox/re_ − *E*_(Fc/Fc_^+^_)_ + 4.8] eV, respectively. Energies of S_1_ (*E*_S1_) and T_1_ (*E*_T1_) and the difference between S_1_ and T_1_ (*ΔE*_ST_) were calculated from their absorption and emission spectra.

### 3.3. Thermal Property Analysis

The glass transition temperature (*T*_g_) was determined from the second heating scan. Thermogravimetric analysis (TGA) measurements were undertaken with a NETZSCH STA 449C instrument. The thermal stability of the samples under nitrogen atmosphere was determined by measuring their weight loss (5%) when heating at a rate of 10 °C min^−1^ from 25 to 500 °C.

### 3.4. Absorption and Emission

UV–visible absorption spectra were recorded on a SHIMADZU UV-1750 spectrometer. Photoluminescence (PL) spectra were measured using a Horiba JobinYvon FluoroLog-3 spectrometer platform.

### 3.5. Computational Analysis

All density functional theory (DFT) and time-dependent density functional theory (TDDFT) calculations were carried out using Gaussian 09 software. Geometry optimizations were performed for S0 and T1 using density functional theory with the B3LYP function. In addition, 6-31G(d) basis sets were adopted for all atoms C, H and N.

### 3.6. OLED Fabrication and Characterizations

The ITO glass substrate used in this paper was 32 mm × 32 mm × 0.7 mm, the thickness of the ITO etched on it was 135 nm, its square resistance was ≤15 Ω/sq and its transmittance was ≥86%. Before the device was fabricated, the ITO glass substrate was first cleaned with detergent, and then ultrasonically cleaned in deionized ultrapure water, acetone and isopropanol, and the three ultrasonication processes continued identically for 20 min. Finally, the surface of the ITO glass substrate was treated with an oxygen plasma etching machine to reduce the surface work function and hydrophilicity of the ITO glass substrate. All organic layers were deposited by vacuum evaporation in a vacuum chamber with a pressure of less than 5 × 10^−4^ Pa. The luminescence area was four rectangles of 3.2 mm × 3 mm, and its area was the overlapping electrode and ITO part. The evaporation rates of the organic materials and aluminum were 1.0–2.0 and 3.0–5.0 Ås^−1^, respectively.

A computer-controlled Ocean Optics QE 65 Pro fiber optic spectrometer and Keithley 2400 light source meter measured EL spectra, CIE coordinates and current–voltage–brightness (J–V–L) characteristics in a darkroom at room temperature. The lifetimes of all PHOLED devices were tested with integrating spheres under the same conditions. EQE, CE and PE were measured with the same integrating spheres.

### 3.7. Synthesis and Structure Characterization

#### 3.7.1. Synthesis of m-ICzPBI

To a 200 mL sealed tube were added 2-(3-bromophenyl)-1-phenyl-1*H*-benzimidazole (1.14 g, 3.26 mmol), 2-(4,4,5,5-tetramethyl-1,3,2-dioxacyclopentane-2-yl) indolo[3,2,1-*jk*]carbazole (1.12 g, 3.26 mmol), tetrakis(triphenylphosphine)palladium (185 mg, 0.16 mmol), K_2_CO_3_ (20 mL, 2 M) and THF (20 mL). Nitrogen was used to replace the reaction atmosphere in the bottle. After three times of replacement, the reaction was stirred overnight at 85 ℃. The reaction was monitored using thin layer chromatography (TLC) (PE/EA = 4:1) until completion. After cooling down to room temperature, the mixture was added to a saturated salt solution and extracted with ethyl acetate (20 mL × 3). The obtained organic phase was combined and dried with anhydrous Na_2_SO_4_. After purification by chromatographic column, the white solid was the compound m-ICzPBI (1.403 g, yield 84 %); ^1^H NMR (400 MHz, chloroform-*d*) *δ* 8.13 (d, *J* = 7.6 Hz, 2H), 7.97 (d, *J* = 4.0 Hz, 3H), 7.91 (d, *J* = 8.0 Hz, 3H), 7.79–7.75 (m, 1H), 7.72–7.64 (m, 4H), 7.60–7.55 (m, 2H), 7.51–7.44 (m, 3H), 7.42–7.36 (m, 3H) and 7.32 (d, *J* = 4.0 Hz, 2H); and ^13^C NMR (126 MHz, chloroform-*d*) *δ* 152.40, 143.59, 143.21, 143.02, 139.13, 137.47, 137.31, 136.59, 130.18, 130.10, 130.03, 129.46, 129.16, 128.93, 128.60, 127.81, 127.66, 126.94, 123.49, 123.15, 123.13, 121.87, 119.94, 119.15, 118.55, 112.32 and 110.51. MS (ESI):510.16 [M + H]^+^.

#### 3.7.2. Synthesis of o-ICzPBI

To a 200 mL sealed tube were added 2-(2-bromophenyl)-1-phenyl-1*H*-benzimidazole (417.6 mg, 1.2 mmol), 2-(4,4,5,5-tetramethyl-1,3,2-dioxacyclopentane-2-yl) indolo[3,2,1-*jk*]carbazole (440.7 mg, 1.2 mmol), tetrakis(triphenylphosphine)palladium (70.8 mg, 0.06 mmol), K_2_CO_3_ (15 mL, 2 M) and THF (15 mL). Nitrogen was used to replace the reaction atmosphere in the bottle. After three times of replacement, the reaction was stirred overnight at 85 ℃. The reaction was monitored using thin layer chromatography (TLC) until completion. After cooling down to room temperature, the mixture was added to a saturated salt solution and extracted with EA (20 mL × 3). The obtained organic phase was combined and dried with anhydrous Na_2_SO_4_. After purification by chromatographic column, the white solid was the compound o-ICzPBI (446.3 mg, yield 73%);^1^H NMR (400 MHz, chloroform-*d*) *δ* 8.05–8.01 (m, 1H), 7.96 (d, *J* = 8.1 Hz, 1H), 7.91–7.85 (m, 4H), 7.60–7.52 (m, 4H), 7.42–7.36 (m, 1H), 7.32–7.27 (m, 5H), 7.13–7.04 (m, 2H), 6.93 (d, *J* = 8.0 Hz, 1H), 6.79 (t, *J* = 6.8 Hz, 2H) and 6.18 (d, *J* = 6.0 Hz, 2H); and ^13^C NMR (126 MHz, chloroform-*d*) *δ* 153.43, 143.23, 143.19, 142.91, 138.94, 135.95, 135.62, 135.21, 132.46, 131.00, 130.13, 129.92, 128.68, 127.20, 126.76, 126.69, 125.55, 123.15, 123.03, 122.66, 121.81, 120.36, 119.84, 118.11, 112.17 and 110.31. MS (ESI): 510.13 [M + H]^+^.

## 4. Conclusions

In summary, the structure–property relationships of the basic motifs TPA, PCz and ICz were comparatively investigated. Among these, the ICz moiety, in which all the phenyls are internally cyclized with a carbon–carbon bond, is in a particularly good planarity. This grants ICz with high rigidity and an integral multiple resonance effect. ICz was then proved to have high thermal stability, a large band gap and a balanced carrier (both hole and electron) transport capability so that it is suitable for constructing blue hosts. Two hosts, m-ICzPBI and o-ICzPBI, were successfully synthesized and fully investigated. m-ICzPBI and o-ICzPBI, balanced in electron and hole transport, were incorporated with FIrpic to prepare phosphorescent devices A and B, respectively, by evaporation. Device A, showing better performance, achieved high EQE_max_, PE_max_ and CE_max_ of 13.4%, 24.8 lm W^−1^ and 31.6 cd A^−1^. Device A also showed low roll-off and retained EQE, PE and CE of 20.3 cd A^−1^, 10.6 lm W^−1^ and 8.6% at a luminance of 5000 cd/m^2^. These results indicate the outstanding properties of the ICz moiety with a multiple resonance effect for constructing host materials, which are potential candidates for efficient and durable blue PhOLEDs.

## Figures and Tables

**Table 1:** Molecular energy levels calculated by DFT.

Motif	HOMO (eV)	LUMO (eV)	Eg (eV)	*E*_S1_ (eV)	*E*_T1_ (eV)
TPA	−4.95	−0.30	4.65	3.93	3.18
PCz	−5.33	−0.65	4.68	4.04	3.18
ICz	−5.56	−1.25	4.31	3.68	2.98

**Table 2:** Photophysical properties.

Compound	λ_abs_ ^a^ (nm)	λ_PL_ ^a/b^ (nm)	*E*_S1_ (eV)	*E*_T1_ (eV)	*E*_HOMO_/*E*_LUMO_/E_g_ ^c^ (eV)	*E*_IP_ ^d^ (eV)	*E*_opt_ ^e^ (eV)
m-ICzPBI	275, 287, 374	387/392	3.32	2.83	−5.81/−2.17/3.64	−6.14	3.21
o-ICzPBI	267, 290, 371	382/383	3.35	2.83	−5.68/−2.18/3.50	−6.06	3.25

^a,b^ Absorption peaks (λ_abs_) and emission peaks (λ_PL_) were obtained from the spectra measured in DCM (a) and PMMA (b), respectively. Energies of the lowest singlet (*E*_S1_) and triplet (*E*_T1_) were calculated from the absorption peaks in DCM and emission peaks at 77 K, respectively. ^c^ The energy levels of HOMO (*E*_HOMO_) and LUMO (*E*_LUMO_) were calculated from CV data measured in DMF solution, and the energy gap values were calculated by *E*_g_ = *E*_LUMO-_*E*_HOMO_. ^d^ The ionic potentials (*E*_IP_) were measured by pure solid using IPS-4. ^e^ Optical gap (*E*_opt_) was obtained from absorption edge.

**Table 3:** OLED performance parameters for FIrpic-based OLEDs.

Device	V_on_ (V)	@ max/1000/5000 cd m^−2^			λ_max_ (nm)	CIE (x, y)
		EQE (%)	PE (lm W^−1^)	CE (cd A^−1^)		
A	3.0	13.4/11.2/8.6	24.8/18.3/10.6	31.6/26.3/20.3	475	0.17, 0.40
B	3.0	12.5/10.6/7.8	23.2/10.5/8.8	29.5/25.4/18.5	475	0.17, 0.40

## Data Availability

Not applicable.

## Supplementary Materials

The following supporting information can be downloaded at https://www.mdpi.com/article/10.3390/molecules28135118/s1: Figure S1: ^1^H NMR of m-ICzPBI. Figure S2: ^13^C NMR of m-ICzPBI. Figure S3: ^1^H NMR of o-ICzPBI. Figure S4: ^13^C NMR of o-ICzPBI. Figure S5: LC-MS of m-ICzPBI. Figure S6: LC-MS of o-ICzPBI. Figure S7: TGA of TPA, PCz, ICz, m-ICzPBI and o-ICzPBI. Figure S8: DSC of m-ICzPBI and o-ICzPBI. Figure S9: Cyclic voltammetry curves. Figure S10: Device structure of the PhOLED and functional molecules. Figure S11: Current density–voltage characteristics for hole-only devices and electron-only devices with m-ICzPBI (a) and o-ICzPBI (b).

## Sample Availability

Samples of the compounds are available from the authors.
