# Supplementary material for: Multi-Resonant Indolo[3,2,1-jk]carbazole-Based Host for Blue Phosphorescent Organic Light-Emitting Diodes
Source: Molecules. 2023 Jun 29;28(13):5118. doi: 10.3390/molecules28135118 (PMC10343769; doi:10.3390/molecules28135118)
Supplement: Supplementary file 1 [file molecules-28-05118-s001.zip › molecules-2442770-supplementary.pdf]

Supplementary Information

Multi-resonant indolo[3,2,1-*jk*]carbazole based host for blue phosphorescent organic light-emitting diodes

Xiang Li[†], Peng Yuan[†], Jinyu Song[†], Yu Chang[†], Xueting Jiao[†], Jianfen Zhao[†], Cong Zhang*[†], Wenjuan Li[‡] and Xiao-Chun Hang*[†]

[†] School of Flexible Electronics (Future Technologies), Institute of Advanced Materials, Nanjing Tech University, 30 South Puzhu Road, Nanjing 211816, P.R. China, iamczhang@njtech.edu.cn, iamxchhang@njtech.edu.

[‡] Nanjing Jianuolin Optoelectronics Technology Co. Ltd. 5th Xin Mofan Road, Nanjing, 210009, P.R. China

Contents

General Procedures **3**

Synthesis and Structure Characterization. **5**

^{1}H NMR, ^{13}C NMR and LC-MS Spectra **6**

Thermal and Electroluminescent Properties **9**

Device **11**

General Procedures

Structure Characterization

^{1}H NMR spectra were recorded at 400 MHz on NMR instruments in Chloroform-*d* or DMSO-d_6 solution, ^{13}C NMR spectra were recorded at 126 MHz on Varian Liquid-State NMR instruments in chloroform-*d* solution. Mass spectra were recorded on liquid chromatograph mass spectrometer (LC-MS).

Electrochemical Analysis

Cyclic voltammetry was performed using a CH Instrument 660E electrochemical analyzer under a nitrogen atmosphere in glovebox. Anhydrous DMF was used as the solvent for reduction and oxidation, respectively. 0.1 M tetra(*n*-butyl) ammonium hexafluorophosphate was used as the supporting electrolyte. A silver wire was used as the pseudo reference electrode, a Pt wire was used as the counter electrode, and platinum column was used as the working electrode. The redox or oxidation potentials are reported referring to ferrocenium/ferrocene (Fc^+/Fc). Their HOMO and LUMO energy levels were calculated from the onset of oxidation (E_{ox}) and reduction potentials (E_{re}) according to the equation of EHOMO/LUMO = $-[E_{ox/re} - E_{(Fc/Fc^+)} + 4.8]$ eV, respectively. Energy of S_1 (E_{S1}), T_1 (E_{T1}) and difference between S_1 and T_1 (ΔE_{ST}) are calculated from their absorption and emission spectra.

Thermal Property Analysis

The glass transition temperature (T_g) was determined from the second heating scan. Thermogravimetric analysis (TGA) measurements were undertaken with NETZSCH STA 449C instrument. The thermal stability of the samples under nitrogen atmosphere was determined by measuring their weight loss (5%) when heating at a rate of 10 °C min^{-1} from 25 to 500 °C.

Absorption and Emission

The UV-visible absorption spectra were recorded on a SHIMADZU UV-1750 spectrometer. Photoluminescence (PL) spectra were measured using on a Horiba JobinYvon FluoroLog-3 spectrometer platform.

Computational Analysis

All the density functional theory (DFT) and time-dependent density functional theory (TDDFT) calculations were carried out by using Gaussian 09 software. Geometry optimizations were performed for the S0 and T1 using density functional theory with B3LYP functional. Also, 6-31G(d) basis sets are adopted for all atoms C, H, and N.

OLED Fabrication and characterizations

The ITO glass substrate used in this paper is 32 mm×32 mm×0.7mm, the thickness of the ITO etched on it is 135 nm, its square resistance ≤ 15 Ω/sq, and the transmittance is ≥ 86%. Before the device is fabricated, the ITO glass substrate is first cleaned with detergent, and then ultrasonically cleaned in deionized ultrapure water, acetone and isopropanol, and the three ultrasonication processes continued identically for 20 min. Finally, the ITO of glass substrate surface treated with an oxygen plasma etching machine to reduce the surface work function and hydrophilicity of the ITO glass substrate. All organic layers are deposited by vacuum evaporation in a vacuum chamber with a pressure of less than 5×10^{-4} Pa. The luminescence area is four rectangles of 3.2 mm × 3 mm, and its area is an overlapping electrode and ITO part. The evaporation rates of organic materials and aluminum were 1.0-2.0 and 3.0-5.0 $Ås^{-1}$, respectively.

The computer-controlled Ocean Optics QE 65 Pro fiber optic spectrometer and Keithley 2400 light source meter measure EL spectra, CIE coordinates, and current-voltage-brightness (J-V-L) characteristics in a darkroom at room temperature. The lifetime of all PHOLED devices is tested with integrating spheres under the same conditions. EQE, CE, and PE are measured with the same integrating spheres.

Synthesis and Structure Characterization.

Synthesis of m-ICzPBI. To a 200 mL sealed tube were added 2-(3-bromophenyl)-1-phenyl-1*H*-benzimidazole (1.14 g, 3.26 mmol), 2-(4,4,5,5-tetramethyl-1,3,2-dioxacyclopentane-2-yl) indolo[3,2,1-*jk*]carbazole (1.12 g, 3.26 mmol), tetrakis(triphenylphosphine)palladium (185 mg, 0.16 mmol), K_2CO_3 (20 mL, 2 M), and THF (20 mL). Nitrogen was used to replace the reaction atmosphere in the bottle. After three times of replacement, the reaction was stirred overnight at 85 °C The reaction was monitored with thin layer chromatography (TLC) (PE/EA = 4:1) until completion. After cooling down to room temperature, the mixture was added to saturated salt solution and extracted with ethyl acetate (20 mL × 3). The obtained organic phase was combined, dried with anhydrous Na_2SO_4. After purification by chromatographic column, the white solid is the compound m-ICzPBI (1.403 g, yield 84 %). ^{1}H NMR (400 MHz, Chloroform-*d*) δ 8.13 (d, J = 7.6 Hz, 2H), 7.97 (d, J = 4.0 Hz, 3H), 7.91 (d, J = 8.0 Hz, 3H), 7.79 – 7.75 (m, 1H), 7.72 – 7.64 (m, 4H), 7.60 – 7.55 (m, 2H), 7.51 – 7.44 (m, 3H), 7.42 – 7.36 (m, 3H), 7.32 (d, J = 4.0 Hz, 2H). ^{13}C NMR (126 MHz, Chloroform-*d*) δ 152.40, 143.59, 143.21, 143.02, 139.13, 137.47, 137.31, 136.59, 130.18, 130.10, 130.03, 129.46, 129.16, 128.93, 128.60, 127.81, 127.66, 126.94, 123.49, 123.15, 123.13, 121.87, 119.94, 119.15, 118.55, 112.32, 110.51. MS (ESI):510.16 $[M+H]^+$.

Synthesis of o-ICzPBI. To a 200 mL sealed tube were added 2-(2-bromophenyl)-1-phenyl-1*H*-benzimidazole (417.6 mg, 1.2 mmol), 2-(4,4,5,5-tetramethyl-1,3,2-dioxacyclopentane-2-yl) indolo[3,2,1-*jk*]carbazole (440.7 mg, 1.2 mmol), tetrakis(triphenylphosphine)palladium (70.8 mg, 0.06 mmol), K_2CO_3 (15 mL, 2 M), and THF (15 mL). Nitrogen was used to replace the reaction atmosphere in the bottle. After three times of replacement, the reaction was stirred overnight at 85 °C The reaction was monitored with thin layer chromatography (TLC) until completion. After cooling down to room temperature, the mixture was added to saturated salt solution and extracted with EA (20 mL × 3). The obtained organic phase was combined, dried with anhydrous Na_2SO_4. After purification by chromatographic column, the white solid is the compound o-ICzPBI (446.3 mg, yield 73%).^{1}H NMR (400 MHz, Chloroform-*d*) δ 8.05 – 8.01 (m, 1H), 7.96 (d, J = 8.1 Hz, 1H), 7.91 – 7.85 (m, 4H), 7.60 – 7.52 (m, 4H), 7.42 – 7.36 (m, 1H), 7.32 – 7.27 (m, 5H), 7.13 – 7.04 (m, 2H), 6.93 (d, J = 8.0 Hz, 1H), 6.79 (t, J = 6.8 Hz, 2H), 6.18 (d, J = 6.0 Hz, 2H). ^{13}C NMR (126 MHz, Chloroform-*d*) δ 153.43, 143.23, 143.19, 142.91, 138.94, 135.95, 135.62, 135.21, 132.46, 131.00, 130.13, 129.92, 128.68, 127.20, 126.76, 126.69, 125.55, 123.15, 123.03, 122.66, 121.81, 120.36, 119.84, 118.11, 112.17, 110.31. MS (ESI): 510.13 $[M+H]^+$.

^{1}H NMR, ^{13}C NMR and LC-MS Spectra

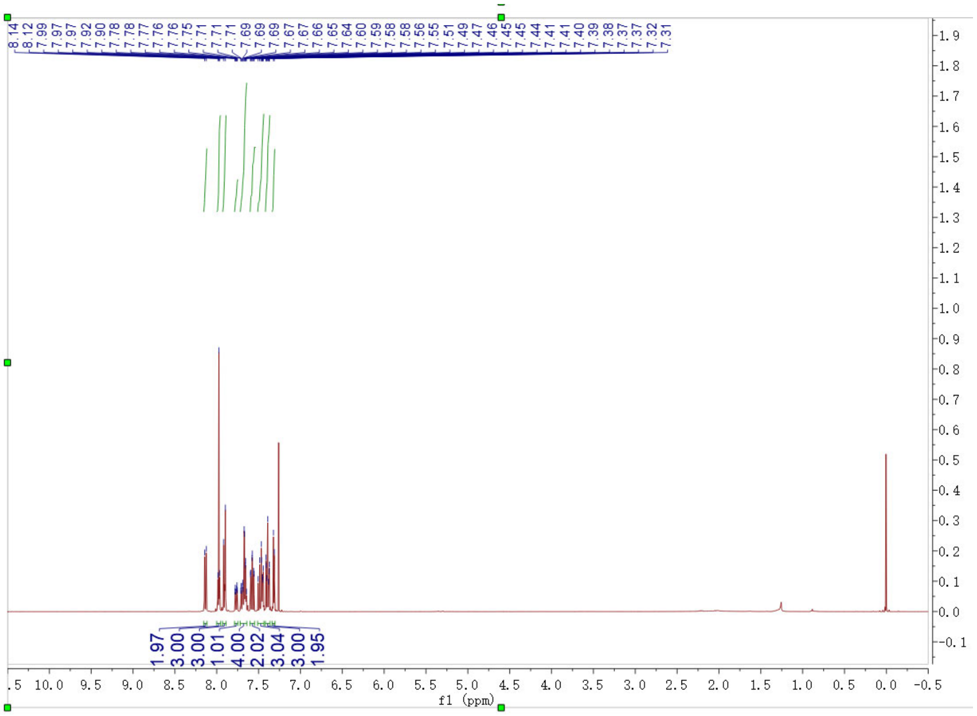

Figure S1. ^{1}H NMR of m-ICzPBI.

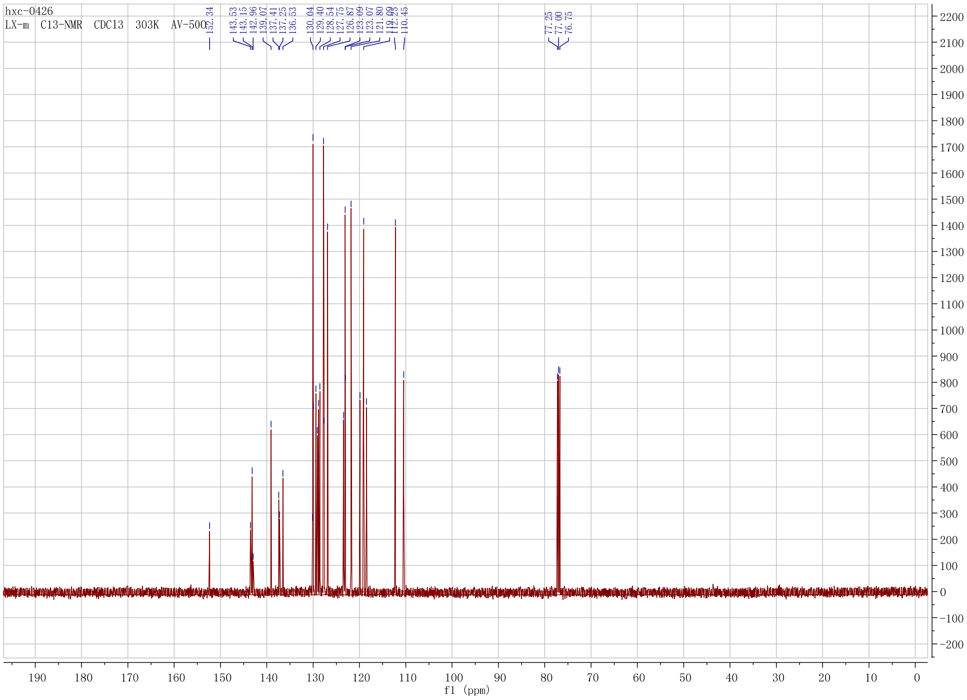

Figure S2. ^{13}C NMR of m-ICzPBI in Chloroform-*d*.

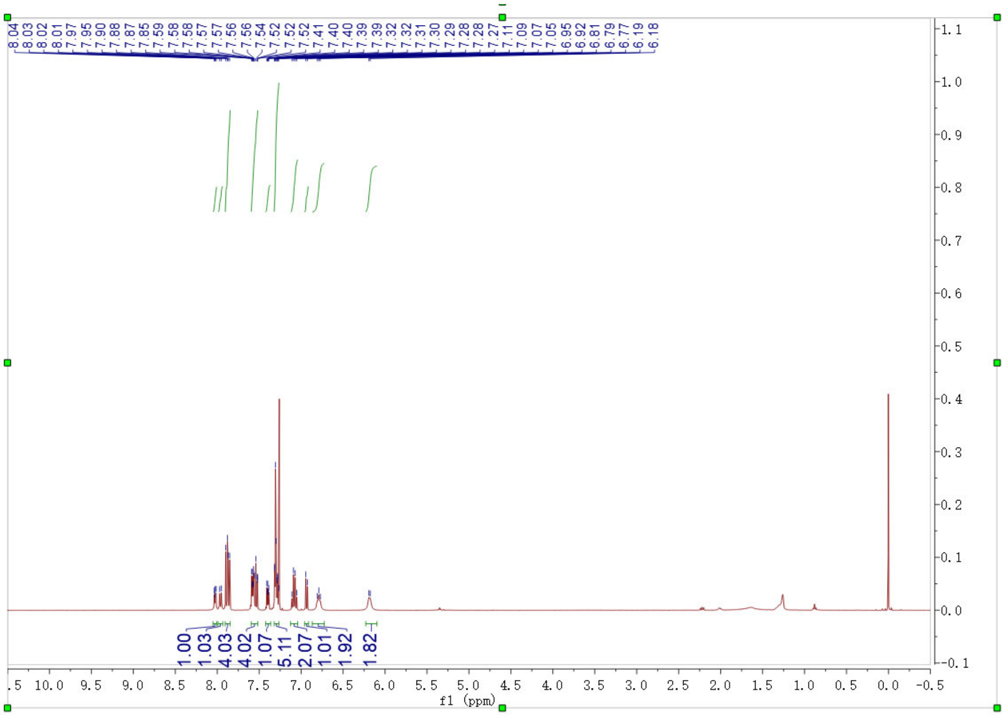

Figure S3. ^{1}H NMR of o-ICzPBI.

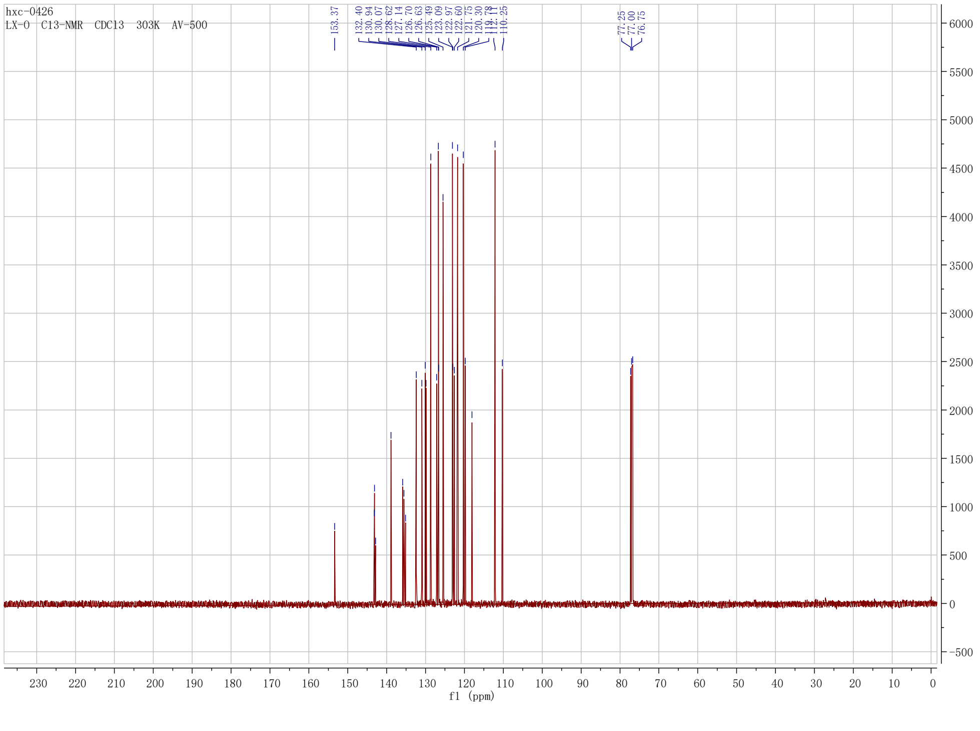

Figure S4. ^{13}C NMR of o-ICzPBI in Chloroform-*d*.

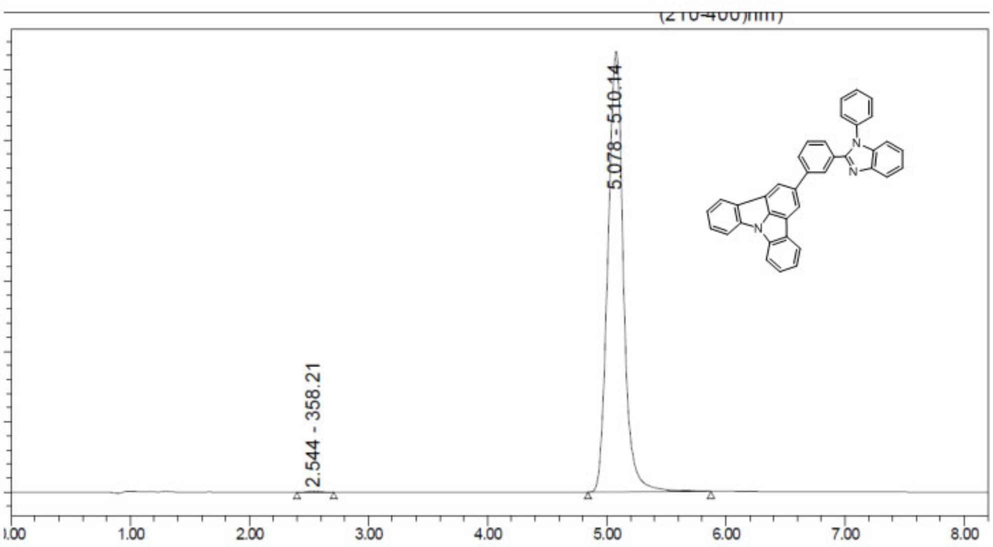

Figure S5. LC-MS of m-ICzPBI.

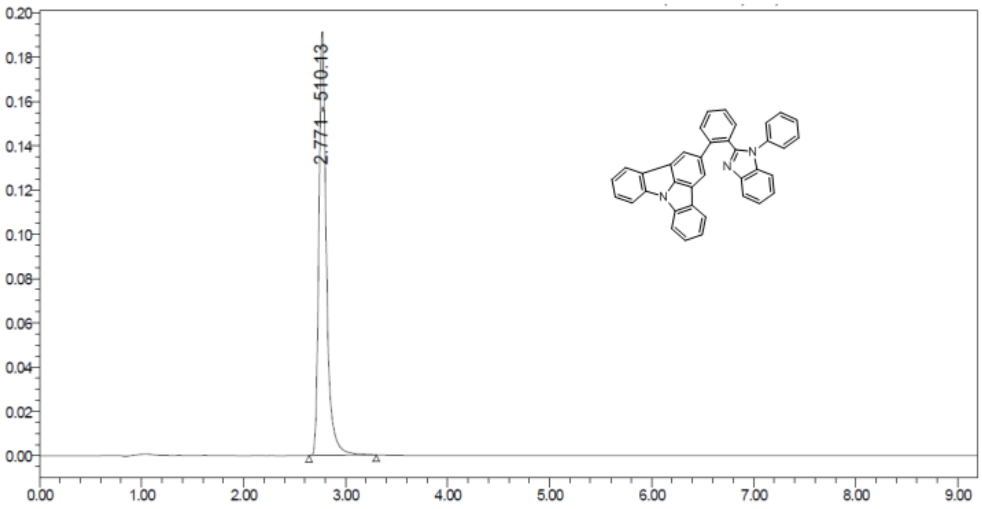

Figure S6. LC-MS of o-ICzPBI.

Thermal and Electroluminescent Properties

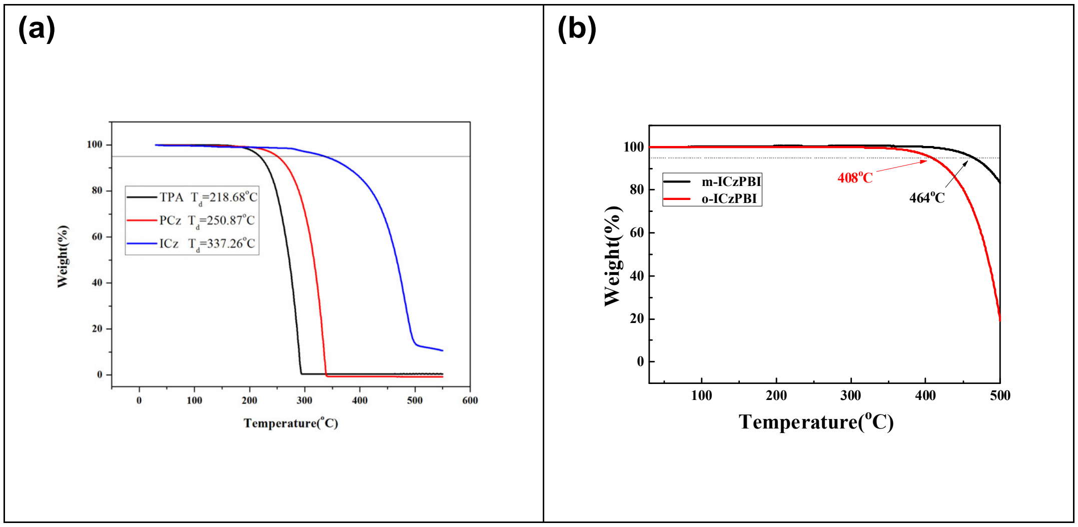

Figure S7. The TGA of TPA, PCz, ICz, m-ICzPBI and o-ICzPBI.

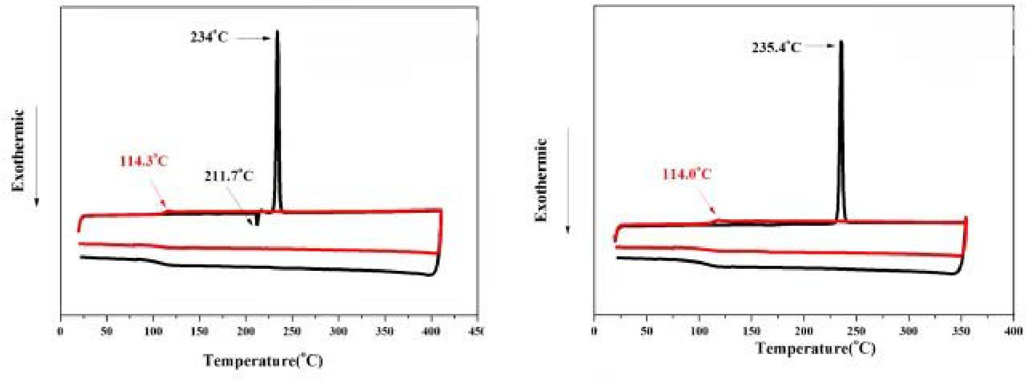

Figure S8. The DSC of m-ICzPBI (left) and o-ICzPBI (right).

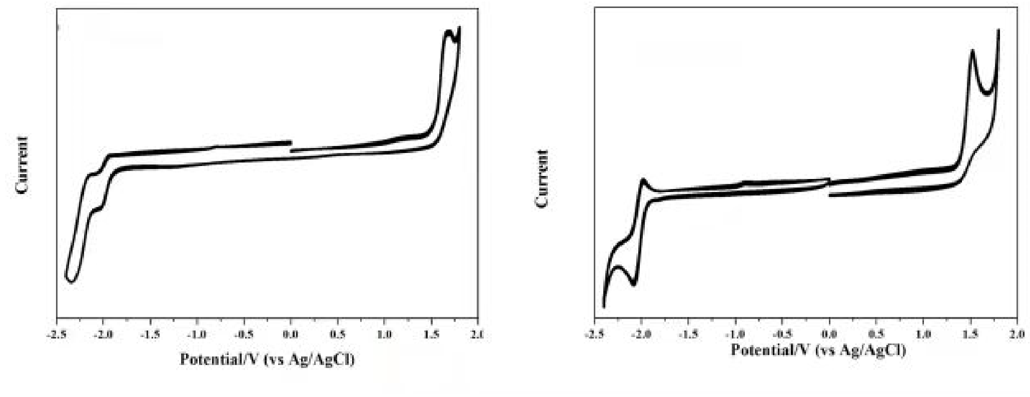

Figure S9. Cyclic voltammetry curves of m-ICzPBI (left) and o-ICzPBI (right).

Device

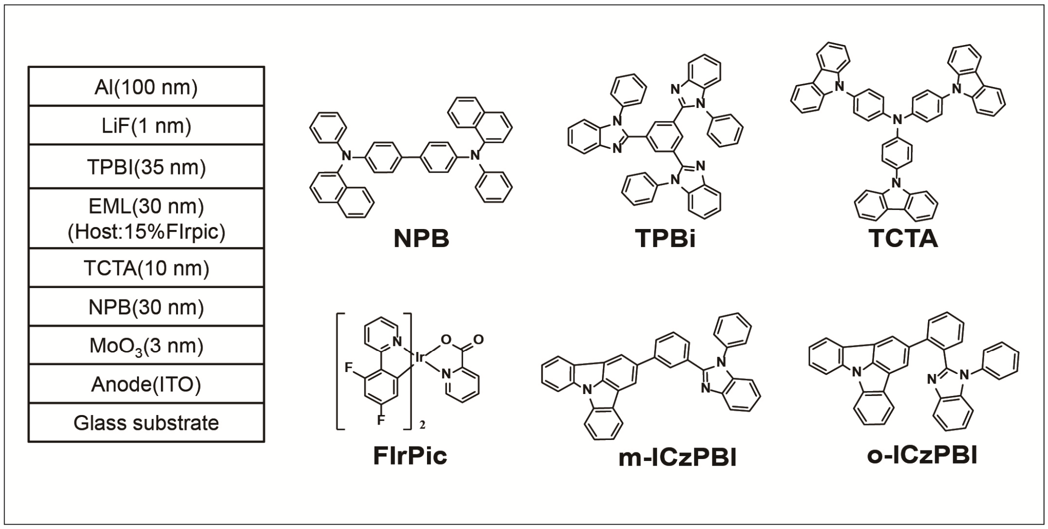

Figure S10. Device structure of the PhOLED and functional molecules.

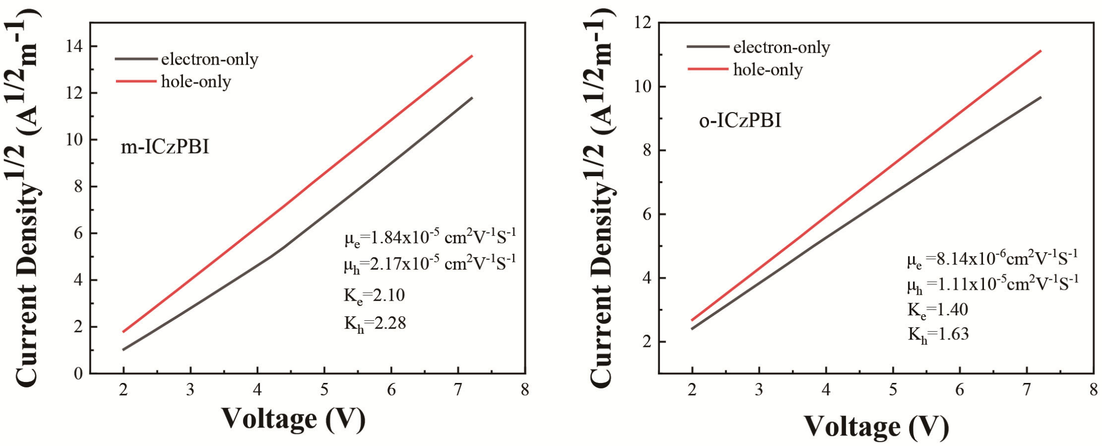

Figure S11. Current density–voltage characteristics for hole-only devices and electron-only devices with m-ICzPBI (a) and o-ICzPBI (b).
